# Effect of roasting and frying treatments on aflatoxins and capsaicinoids content and nutritional profile of green chilies (*Capsicum annum* L.)

**DOI:** 10.1002/fsn3.2966

**Published:** 2022-07-15

**Authors:** Omar Bashir, Vasudha Sharma, Syed Zameer Hussain, Bazila Naseer, Tawheed Amin, Kashif Ameer, Shakeel Ahmad Bhat, Sobiya Manzoor, Isam A. Mohamed Ahmed

**Affiliations:** ^1^ Department of Food Technology and Nutrition Lovely Professional University Phagwara Punjab India; ^2^ Division of Food Science and Technology Sher‐e‐Kashmir University of Agricultural Sciences and Technology, Kashmir Srinagar Jammu and Kashmir India; ^3^ Division of Food Technology Jamia Hamdard New Delhi India; ^4^ Institute of Food Science and Nutrition University of Sargodha Sargodha Pakistan; ^5^ College of Agricultural Engineering, Sher‐e‐Kashmir University of Agricultural Sciences and Technology, Kashmir Srinagar Jammu and Kashmir India; ^6^ Department of Food Science and Technology, Faculty of Agriculture University of Khartoum Shambat Sudan; ^7^ Department of Food Science and Nutrition, College of Food and Agricultural Sciences King Saud University Riyadh Saudi Arabia

**Keywords:** aflatoxins, capsaicinoids, frying, green chilies, nutritional profile, roasting

## Abstract

This study was conducted to assess the effect of two thermal treatments, viz. roasting and pan frying (deep frying), on nutritional profile, aflatoxin, and capsaicin content in green chilies. Green chilies were subjected to roasting and frying to reduce the aflatoxin contamination, besides retaining their pungency and nutritional profile. Reversed‐phase HPLC was employed to determine the levels of aflatoxins B1, B2, G1, and G2 in thermally treated and control samples. The proximate compositions of roasted and fried chili samples were significantly (*p* ≤ .05) different from raw chili (control), except ash content. Vitamin A levels decreased significantly (*p* ≤ .05) during roasting and were undetected in fried chili samples. Likewise, vitamin C was undetected in both roasted and fried chili samples. Significantly decreasing (*p* ≤ .05) trends were noticed in capsaicinoids viz. capsaicin and dihydrocapsaicin contents including Scoville Heat Units (SHU) during roasting and frying. However, retention of capsaicinoids was higher in roasted chilies (730.00 ± 4.90 mg/kg) than fried samples (502.56 ± 5.10 mg/kg). The levels of all the four major aflatoxins (AFs)‐ AFB1, AFB2, AFG1, and AFG2 recorded in control were much higher than the limits prescribed by the European Union for spices. Both thermal treatments (roasting and frying) employed proved to be effective in reducing aflatoxins like AFB2, AFG1, and AFG2 in chilies to below the prescribed limits, while as the level of AFB1 was reduced below the limits by only the frying method. This study therefore indicated the substantial impact of frying on aflatoxins.

## Graphical abstract

Effect of thermal treatments on: (a) capsaicin, (b) dihydrocapsaicin, and (c) SHU values of chili samples. Capsaicin (mg/kg), dihydrocapsaicin (mg/kg), and SHU values of control and thermally treated samples indicate a significant (*p* < .05) decrease in capsaicin content on roasting and frying.

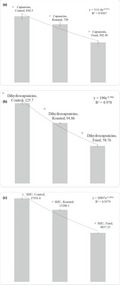

## 1 INTRODUCTION

Green chili (*Capsicum annum* L.), also known as “wonder spice,” belongs to the Solanaceae family. It is mainly used as an ingredient in food processing for color, flavor, and aroma. India accounts for a 35% share (13.76 million tons) of total world chili production and is the leading chili producing country in the world, followed by China (Geetha & Selvarani, 2017). Indian chili holds a significant position in the international market owing to its color and pungency (Geetha & Selvarani, 2017). Green chili, either as fresh or in powder form, is added in a meager amount to foods to enhance their palatability. It contains vitamins (A and C), carotenoids, antioxidants, and minerals in appreciable amounts (Emmanuel‐Ikpeme et al., 2014). The peculiar pungent sensation of green chilies is attributed to the capsaicin, which triggers a hot sensation in the mouth. There is well‐documented evidence that chilies have analgesic, anti‐inflammatory, thermogenic, and antilithogenic properties, besides being beneficial for diabetic neuropathy, gastrointestinal disorders, arthritis, psoriasis, and cardiovascular diseases (Backonja et al., 2008).

Although chili is shelf stable, global concerns are on the rise over its aflatoxin (AF) contamination (Frisvad et al., 2006) and hence scientific interventions are direly needed to promote or regulate the import/export of chilies across the international market. Aflatoxins (AFs) are natural mutagenic mycotoxins produced as secondary metabolites by fungus, especially *Aspergillus parasiticus* and *Aspergillus flavus*, under favorable conditions of growth (Paterson & Lima, 2009). The aflatoxins are classified as Aflatoxin B_1_, B_2_, G_1_, and G_2_ based on characteristic blue and green fluorescence produced under UV light (Reddy & Waliyar, 2012). These four types of aflatoxins are considered Class‐I carcinogens by the International Agency for Research on Cancer (IARC). Among aflatoxins, the highest toxicity is exhibited by Aflatoxin B_1_ (AFB1) (Towner et al., 2000) and is frequently found in chilies. Consumption of aflatoxin contaminated foods leads to aflatoxicosis, which under acute conditions suppresses the immune system and causes death. Aflatoxin contamination in chilies can be either field‐infested or can occur when improper or unhygienic conditions are followed during its harvesting, cleaning, transportation, packaging, and storage. Further, climatic conditions in the Indian subcontinent are favorable for the proliferation of fungus species associated with aflatoxin production (Turner et al., 2009).

It is quite arduous to prevent contamination of chilies by aflatoxins. However, degradation and/or removal of aflatoxins by efficient means seems to be a proper approach to cope with the stringent regulatory quality standards for export of chilies. Several biological and physicochemical methods have been investigated in this regard, but only a few have been found acceptable due to the limitations of large‐scale applicability, cost, environmental pollution, efficacy, safety, and others (Pankaj et al., 2018). Use of heat treatment to reduce aflatoxins in various commodities has been well demonstrated (Ginting et al., 2018; Rastegar et al., 2017) but very limited methods of thermal processing have been tested so far to lower the contamination of chilies by aflatoxins (Singh & Cotty, 2017). However, degradation of aflatoxins by heat treatment depends on various factors such as temperature, time, food composition, and medium of treatment. Thus, it becomes imperative to assess the effect of different thermal methods of processing on aflatoxin content of chilies. The present study was therefore conducted to evaluate two thermal processing methods, viz. roasting and frying, as a means to reduce the aflatoxin contamination in green chilies, besides retaining their pungency and nutritional profile.

## 2 MATERIALS AND METHODS

### 2.1 Raw material and chemicals

Fresh green chilies were procured from local vegetable markets of Tughlakabad Extension, New Delhi, India. All of the chemicals used were of analytical grade from Sigma‐Aldrich, Inc. and Merck.

### 2.2 Sample preparation

Green chilies were ground in a grinder (Bajaj GX series) and the ground sample, after thorough mixing, was stored in sealed polyethylene bag under refrigerated conditions (4 ± 2°C) for further processing and analysis.

### 2.3 Processing of green chilies

Chilies were subjected to roasting and frying. Chilies without any treatment were considered as control.

### 2.4 Roasting

A hot air oven having a 20 × 14 × 10 inch chamber (Remi RDHO series) was preheated to 130–135°C. After temperature equilibration (130–13°C), the prepared chili samples were introduced into the drying chamber and spread in a single layer (thickness 0.5 inch) on an aluminum foil. A stainless steel plate was kept between the heating elements and the foil to prevent the direct radiation exposure and to ensure a more even temperature distribution. The sample was roasted at 130–135°C for 10–12 h.

### 2.5 Frying

Briefly 100 ml mustard oil was poured on a frying pan that was placed on a hot plate (GKST 300Z, THIELMANN) for 2 min. Ten grams of chili paste sample was then placed on the pan and frying was done at a temperature of 260°C for 10 min with continuous stirring. After this, the chili samples were taken out of the oil and paced on blotting paper to absorb the extra oil from chili samples.

### 2.6 Physicochemical analysis

Standard AOAC (2005) methods were used to estimate the moisture (method 930.15), ash (method 942.05), fat (method 948.22), protein (method 981.10), carbohydrate (method 995.13), and crude fiber (method 978.10) contents in control, roasted and fried green chili samples.

### 2.7 Estimation of vitamin A

Vitamin A was estimated by a High‐Performance Liquid Chromatography (HPLC) method. Control, roasted, and fried chili paste samples (5 g each) were weighed in a flat‐bottomed flask. Then, 40 ml of ethanol and 10 ml of 50% potassium hydroxide were added to it. To prevent oxidative degradation of the vitamin A, a pinch of pyrogallate was added. It was then refluxed for 45 min at 95 °C followed by cooling. Water (40 ml) and ethanol (20 ml) were then added. Extraction of vitamin A was done using petroleum ether and hexane in a separating flask. The mixture after filtering using Whatman filter paper No.1 (GE Healthcare) was dried under nitrogen flux at 41°C. The residue was then reconstituted with methanol (1 ml), vortexed, filtered through 0.45‐μm membrane, and then transferred to liquid chromatography vials for analysis by HPLC (Agilent Technologies 1200 series). The mobile phase used was methanol: water. The detection was done using photodiode array (PDA) detector at 325 nm (AOAC, 1995) and compared with that of an authentic standard of vitamin A. The column used was Agilent InfinityLab Poroshell 120 HILIC‐Z, 2.1 × 100 nm, 2.7 μm (p/n 685,775–924).

### 2.8 Estimation of vitamin C

Vitamin C estimation was done using Atlantis T3 C18 column (Waters) attached to Agilent model 1200 HPLC (Agilent Technologies) system. Briefly, 5 g each of control, roasted, and fried chili sample were weighed, and 25 ml of water used as mobile phase was added to it. After manual shaking, it was subjected to sonication for 5 min followed by centrifugation (Remi) at 4500 × *g* for 10 min. The supernatant obtained was filtered through 0.45‐μm membrane filter for analysis (AOAC, 1995) by HPLC. Water was used as the mobile phase and a flow rate of 0.6 ml/min was maintained. The detection of vitamin C was performed using PDA detecting system at 230 nm and compared with that of authentic standard of vitamin C.

### 2.9 Estimation of aflatoxins

Reversed‐phase HPLC (Agilent, 1260) was employed to determine the levels of aflatoxinsB_1_, B_2_, G_1_, and G_2_ in thermally treated and control samples. Carbon‐18 (C18) analytical column (X‐Bridge‐Waters) was used in this study. The mobile phase consisted of acetonitrile: water (80:20, v/v) at a flow rate of 1 ml/min. The column temperature was maintained at 40°C. Briefly, 2 g chili sample was weighed in a tarson tube and 10 ml of acetonitrile: water (80:20) with 0.1% formic acid was added to it. It was then vortexed for 1 min followed by shaking mechanically for 20 min. After adding 1 g of NaCl, the whole mixture was centrifuged at 4500 × *g* for 10 min. This was followed by filtration through Whatman filter paper No.1. The filtrate was defatted with n‐hexane and dichloromethane for extraction of aflatoxins (AFs). It was again filtered through a 0.45‐μm membrane filter from which 200 μl was collected. The volume was then made up to 1 ml by methanol. The mixture was vortexed and syringe filtered through 0.45‐μm membrane before transferring to liquid chromatography vials. All of the samples were then analyzed using HPLC MS/MS triple quadrupole system (AOAC, 2007).

### 2.10 Estimation of capsaicinoids

The major capsaicinoids (capsaicin and dihydrocapsaicin) were analyzed using HPLC (HP 1100 Agilent Technology) installed with a C18 column (X‐Bridge) (5 μ, 50 × 4.6 mm) and a UV detector at 284 nm. Briefly, 20 g sample and the mobile phase (acetonitrile: water) mixture was refluxed at 60–65°C for 5 h and then cooled. The refluxed mixture was centrifuged at 4500 × *g* and vacuum‐filtrated to obtain a clear supernatant. Acetone was evaporated to obtain the crude extract, which was then diluted in 50 ml methanol and filtered through 0.45‐μm syringe filter. Then, 2 ml of the supernatant was transferred to a vial test tube for analysis (AOAC method No. 995.03, 1995). The analysis was carried out at 25°C and the mobile phase used was a mixture of acetonitrile and water (65:35 v/v) and the flow rate was 1 ml/min. The capsaicin and dihydrocapsaicin in the chili samples were identified and quantified by comparison with 98% pure capsaicin and 90% pure dihydrocapsaicin standard compounds (Sigma‐Aldrich, Inc.). Standard curves were prepared using serial dilutions of 25, 50, 100, 200, and 400 mg/L for capsaicin and 12.5, 25, 50, 100, and 200 mg/L for dihydrocapsaicin.

### 2.11 Determination of Scoville heat units (SHU)

The method prescribed by Todd (Todd Jr et al., 1977) was followed to estimate the SHU. SHU was calculated by multiplying the threshold pungency of capsaicin and dihydrocapsaicin with their respective concentrations (Equation 1) (Orellana‐Escobedo et al., 2013).

(1) $$\mathrm{SHU}=\left[\left(\%\text{capsaicin}\times 16.1\right)+\left(\%\text{Dihydrocapsaicin}\times 16.1\right)\right]$$

### 2.12 Statistical analysis

The observations were recorded as the average of triplicate (*n* = 3) samples. One‐way ANOVA was employed to test the significance of difference (*p* ≤ .05) using SPSS software. The differences between the means were compared using the Duncan Multiple Range test. The data were presented as mean ± standard deviation.

## 3 RESULTS AND DISCUSSION

### 3.1 Chemical composition

Chemical composition of control and thermally treated (roasted and fried) green chilies are shown in Table 1. Thermal treatments significantly (*p* < .05) affected the chemical composition of chilies except ash contents (*p* ≤ .05). Moisture content reduced significantly from 87.61 ± 1.50% in control to 23.45 ± 2.30% in fried and 2.05 ± 0.20%% in roasted chilies (Table 1). Exposure of chilies to high temperature (130–135°C) for long duration (10 h) during roasting caused dehydration in chilies, which resulted in lowering of moisture content in roasted chili samples. The higher moisture content of fried chilies than that of roasted ones may be due to lower frying time (10 min). Locatelli et al. (2010) have also demonstrated the significant decrease in moisture content in hazelnuts after prolonged roasting. However, no significant (*p* > .05) difference was recorded in ash content of chilies after roasting or frying (Table 1). Similar results were reported by Arinola and Adesina (2014) for African walnuts subjected to roasting and other heat treatments. The fat content of fried green chilies (67.69 ± 0.07%) was significantly higher than the control (14.90 ± 0.03%) and roasted green chilies (17.22 ± 0.02%) (*p* ≤ .05) (Table 1). This increase in fat content in fried chilies may be because of the absorption of oil by chilies during frying. Our results corroborated well with the results of Hwang et al. (2012) for red pepper. The protein content in control was 23.44 ± 0.10%, which reduced significantly (*p* ≤ .05) to 19.52 ± 0.12% and 19.52 ± 0.12% in roasted and fried samples, respectively. During roasting and frying, proteins undergo thermal degradation due to high temperature, which causes denaturation of proteins. El‐Beltagi (2011) has also demonstrated a decrease in protein content of Egyptian peanuts after subjecting to roasting treatment. Total carbohydrate content decreased significantly (*p* ≤ .05) in chilies after roasting and frying, although the decrease was higher in fried chilies than in roasted ones. Any thermal treatment approximately at about 170°C or more breaks down large and complex carbohydrates into simple sugars. Under roasting conditions, these sugars participate in Maillard reactions and are susceptible to caramelization, which explains the slight decrease of carbohydrate content in roasted chilies. However, sugars moved from chilies into the frying medium‐oil thus the loss of carbohydrates with frying is justified (Gouado et al., 2011). Bahado‐Singh et al. (2006) also reported similar findings for thermally treated carbohydrate‐rich foods. Crude fiber contents of green chilies increased significantly (*p* ≤ .05) from 18.50 ± 0.08% (control) to 26.44 ± 0.19% (roasted) and decreased significantly (*p* ≤ .05) to 9.66 ± 0.03% in fried chili samples (Table 1). Similar results have been reported by Singh et al. (2018) while evaluating the effect of roasting on functional and phytochemical characteristics of finger millet. The loss of crude fiber in fried chilies could be attributed to leaching of soluble fibers from green chilies into frying medium (oil). More or less similar results were reported by Ramasawmy et al. (1999) for potato‐based products. Green chilies contain an appreciable content of ascorbic acid and vitamin A. Ascorbic acid in green chilies was recorded as 135.7 ± 2.5 mg/kg. Since ascorbic acid is very sensitive to thermal processes, its structure degrades easily at higher temperatures (Lee & Kader, 2000). During thermal processing, ascorbic acid is oxidized to dehydroascorbic acid which further gets hydrolyzed to 2, 3‐diketogluconic acid besides the production of other polymeric compounds (Gregory, 1996) thereby decreasing the vitamin C content in roasted and fried chili (Table 1). Thermal degradation of ascorbic acid has also been observed by Miglio et al. (2008). The vitamin A content in fresh green chilies was estimated to be 20.71 ± 1.43 mg/kg and after roasting it was decreased to 3.62 ± 0.63 mg/kg. Due to the low thermal stability of vitamin A, roasting might have altered its physical state (Oke et al., 2018) which could have reduced the vitamin A during roasting. However, in the case of fried chili, vitamin A could not be detected, possibly due to its fat soluble nature. Vitamin A might have leached out of the green chili into the frying medium (oil), thus was not detected in fried chilies (Akhtar et al., 2012). Several other authors have also confirmed the degradation of vitamin A at a temperature range of 130–140°C (Chen et al., 1995; Oke et al., 2018) and complete loss in frying (Omotosho, 2015). Similar results were also demonstrated for vitamin C and vitamin A in various studies involving thermal processing (Omotosho, 2015; Kadakal et al., 2018).

**TABLE 1:** Chemical composition of control, roasted, and fried chili samples

Sample	Moisture (%)	Ash (%)	Fat (%)	Protein (%)	Carbohydrate (%)	Crude fiber (%)	Vitamin A (mg/kg)	Vitamin C (mg/kg)
Control	87.61 ± 1.50	10.89 ± 0.04	14.90 ± 0.03	23.44 ± 0.10	32.20 ± 0.09	18.50 ± 0.08	20.71 ± 1.43	135.7 ± 2.5
Roasted	2.05 ± 0.20	13.13 ± 0.19	17.22 ± 0.02	19.52 ± 0.12	24.42 ± 0.12	22.02 ± 0.96	3.62 ± 0.63	ND
Fried	23.45 ± 2.30	4.52 ± 0.06	67.69 ± 0.07	7.13 ± 0.08	10.98 ± 0.06	9.66 ± 0.03	ND	ND
C.D (*p* < .05)	.269	.436	.185	.309	.343	.273	.089	1.29

*Note*: Values are the average of triplicate (*n* = 3) and are presented as Mean ± SD. Values of ash, fat, protein, carbohydrates, and crude fiber are given on a dry weight basis.

Abbreviation: NS, Non significant.

### 3.2 Effect of roasting and frying on aflatoxins

Four major aflatoxins, viz. B_1_, B_2_, G_1_, and G_2_, which are harmful for human health, were focused in the present study. In control, aflatoxins‐AFB1, AFB2, AFG1, and AFG2 were recorded as 52.61 ± 3.3, 51.34 ± 2.8, 49.76 ± 2.2, and 53.45 ± 3.1 μg/kg, respectively. These reported values were much higher than the maximum permissible limits of aflatoxins in spices. The maximum tolerable limit for total aflatoxins in spices has been established as 10 and 5 μg/kg for AFB1 by the European Union (EU, 2010). Aflatoxin levels reduced significantly (*p* < .05) in both the treatments – roasting and frying. In roasted chilies, the levels of AFB1, AFB2, AFG1, and AFG2 were recorded as 8.67 ± 2.7, 7.90 ± 2.1, 4.23 ± 1.5, and 6.76 ± 2.5 μg/kg, respectively, whereas in fried chilies, the levels were recorded to be 4.93 ± 2.3, 6.35 ± 1.7, 1.05 ± 0.6, and 2.79 ± 1.6 μg/kg, respectively. Although the aflatoxin levels were much lower in fried chilies than in the roasted, the values recorded for AFB2, AFG1, and AFG2 in both roasted as well as fried chilies were within the permissible limits (10 μg/kg). In contrast, the AFB1 level recorded in fried chilies was within the permissible limit (5 μg/kg), whereas in case of roasted chilies, it was higher than the permissible limits, although nonsignificant (*p* > .05) (Table 2).

**TABLE 2:** Aflatoxin levels in control, roasted, and fried chili samples

Sample	Aflatoxin B1 (μg/kg)	Aflatoxin B2 (μg/kg)	Aflatoxin G1 (μg/kg)	Aflatoxin G2 (μg/kg)
Control	52.61 ± 3.30	51.34 ± 2.80	49.76 ± 2.20	53.45 ± 3.10
Roasted	8.67 ± 0.70 (−83.52)	7.90 ± 0.10 (−84.61)	4.23 ± 0.50 (−91.50)	6.76 ± 0.50 (−87.35)
Fried	4.93 ± 0.30 (−90.63)	6.35 ± 0.70 (−87.63)	1.05 ± 0.06 (−97.88)	2.79 ± 0.06 (−94.78)
C.D (*p* < .05)	.165	.085	.295	.167

*Note*: The negative values in parenthesis indicate the percentage decrease compared to initial values of control. Values are the average of triplicate (*n* = 3) and are presented as Mean ± SD.

During roasting, the levels of AFB1, AFB2, AFG1, and AFG2 levels in chilies reduced by 83.52%, 84.61%, 91.50%, and 87.35%, while as during frying, their levels reduced by 90.63%, 87.63%, 97.88%, and 94.78%, respectively, in relation to their initial values recorded in the control (Table 2). Aflatoxins are thermolabile constituents, which undergo pyrolysis and subsequent reactions with other components at high temperatures. This further leads to their decomposition during thermal processing (Siruguri et al., 2018). Overall, AFG1 and AFG2 aflatoxins degraded more than AFB1 and AFB2. The underlying reason could be the presence of two ether linkages in G series making them more prone to cleavage and disintegration upon thermal treatments (Ogunsanwo et al., 2004). Hamada and Megalla (1982) and Ogunsanwo et al. (2004) have also observed similar findings in roasted soya beans and Nigerian peanuts, respectively.

### 3.3 Effect of roasting and frying on capsaicinoids content and Scoville heat units (SHU)

**FIGURE 1:**
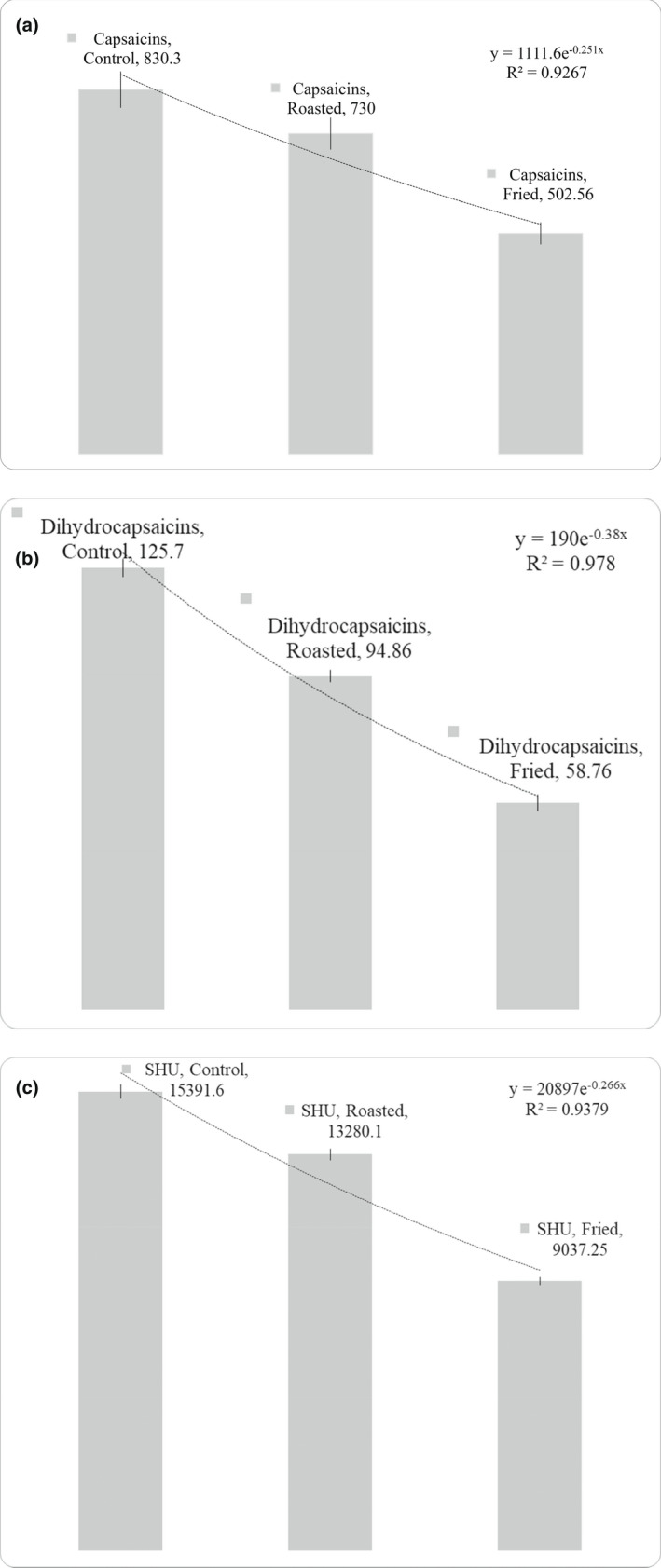
Effect of thermal treatments on: (a) capsaicin, (b) dihydrocapsaicin, and (c) SHU values of chili samples. Capsaicins (mg/kg), dihydrocapsaicin (mg/kg), and SHU values of control and thermally treated samples indicating a significant (*p* < .05) decrease in capsaicin content on roasting and frying (the error bars on each graph represent the standard deviation)

Capsaicinoids include the five analogous constituents, viz. capsaicin, dihydrocapsaicin, nordihydrocapsaicin, homodihydrocapsaicin, and homocapsaicin associated with the pungent sensation in chilies. Among these, capsaicin and dihydrocapsaicin are the most pungent and good predictors of pungency in chilies since they make up 90% of the total capsaicinoids (Reilly et al., 2001). The capsaicin content in control was significantly (*p* < .05) higher (830.30 ± 5.30 mg/kg) than dihydrocapsaicin (125.70 ± 2.50 mg/kg) (Figure 1a,b). Kraikruan et al. have also reported higher capsaicin content than dihydrocapsaicin in various Thai chili cultivars. Compared to the control, both the capsaicin and dihydrocapsaicin decreased significantly (*p* ≤ .05) after thermal treatments (roasting and frying) with more decrease in frying than roasting. The reduction of capsaicin during frying was 39.47%, while, as during roasting, it was only 12.08%. Likewise, during frying, dihydrocapsaicin decreased to 53.25%, whereas during roasting, it decreased by 24.53%. From an initial value of 830.30 mg/kg in control, the capsaicin content decreased to 730.00 ± 4.90 mg/kg during roasting and 502.56 ± 5.10 mg/kg during frying. On the other hand, dihydrocapsaicin decreased to 94.86 ± 1.70 mg/kg during roasting and 58.76 ± 2.40 mg/kg during frying from an initial value of 125.70 ± 2.50 mg/kg in control (Figure 1b). Capsaicinoid structures are degraded during thermal treatment. The alkyl group attached to the amide group of capsaicinoids cleaves during thermal treatment, thereby yielding different products. This cleavage and subsequent oxidation results in the formation of vanillin, which breaks down into phenols (Henderson & Henderson, 1992). A similar decreasing trend in capsaicinoids content has been reported by Arifin and Djaeni (2018) in thermally treated red pepper. Topuz and Ozdemir (2004) have also observed the decrease in capsaicinoids up to 100% in Turkish peppers and Indian Thai when cooked. The significant differences (*p* ≤ .05) in capsaicin and dihydrocapsaicin contents of control, roasted, and fried chilies were reflected in their SHU values as well.

The Scoville scale was used for estimating the intensity of heat in chilies. The scale measures the pungency in terms of spiciness or heat of chilies and other spicy foods. Based on the concentration of capsaicinoids, it is recorded in Scoville Heat Units (SHU). The SHU values for control, roasted, and fried chilies were calculated as 15391.60, 13280.10, and 9037.25 from their respective capsaicin and dihydrocapsaicin values (Figure 1c). The SHU scale given by Weiss (2002) was used to measure the pungency of the samples as: nonpungent with SHU value of 0–700, mild pungent with 700–3000 SHU, moderate pungent with 3000–25,000 SHU, highly pungent with 25,000–70,000 SHU, and very high pungent with SHU value of greater than 80,000. According to this scale, control as well as thermally treated chilies (roasted and fried) could be classified as moderately pungent.

## 4 CONCLUSIONS

Contamination of aflatoxins in chilies is potentially threatening to human health, taking into account the abundant use in food preparations. Therefore, exploring different methods that can not only reduce such contamination but also retain the nutritional profile of chilies are highly recommended. In this regard, the influence of two thermal methods, namely roasting and frying, on aflatoxins, capsaicinoids and chemical composition of chilies was examined. The study confirmed that both roasting and frying methods were effective in maintaining the pungency and reducing the contamination of aflatoxins like AFB2, AFG1, and AFG2 below the permissible limits. However, only frying could reduce the level of AFB1 below the permissible limit. The International Agency for Research on Cancer has included AFB1 among the list of cancer causing agents. Frying is suggested as a more appropriate method than roasting to bring aflatoxins within the safe limits in chilies. The outcome of this study will be handy information to food industries for reducing the aflatoxins in chilies below the permissible limits besides maintaining their pungency. However, frying affected the nutritional profile of chilies, particularly vitamins A and C, which could not be retained in fried chilies. Therefore, further research is needed to optimize the frying procedure for green chilies so as to retain their nutritional profile, particularly vitamins A and C.

## CONFLICT OF INTEREST

The authors declare that they have no known competing interests.

## Data Availability

The data used to support the findings of this study are available from the corresponding author upon request.
